# Comparison of Soaking Corms with Moringa Leaf Extract Alone or in Combination with Synthetic Plant Growth Regulators on the Growth, Physiology and Vase Life of Sword Lily

**DOI:** 10.3390/plants9111590

**Published:** 2020-11-17

**Authors:** Faisal Zulfiqar, Adnan Younis, Patrick M. Finnegan, Antonio Ferrante

**Affiliations:** 1 Institute of Horticultural Sciences, University of Agriculture, Faisalabad 38040, Pakistan; adnanyounis@uaf.edu.pk; 2 School of Biological Sciences, University of Western Australia, 35 Stirling Highway, Perth, WA 6009, Australia; patrick.finnegan@uwa.edu.au; 3 Department of Agricultural and Environmental Sciences, Università degli Studi di Milano, 1 Via Celoria 2, 20133 Milano, Italy; antonio.ferrante@unimi.it

**Keywords:** cut flowers, gladiolus, gibberellic acid, photosynthetic activity, plant biostimulant, sustainable agriculture, salicylic acid, vase life

## Abstract

Gladiolus is in demand worldwide as a cut-flower or landscaping plant, because of its superior commercial and ornamental value. Application of plant-based biostimulants has gained interest in the horticulture industry as an innovative and promising approach to ensure enhanced and sustainable yields along with better product quality. The influence of pre-plant corm soaks supplemented to 5% (*v/v*) with an aqueous extract from *Moringa oleifera* leaves (MLE) either alone or in combination with 50 mg/L salicylic acid (SA) or 50 mg/L gibberellic acid (GA) on the vegetative, physiological, and ornamental characteristics of potted gladiolus (*Gladiolus grandiflorus*) was investigated. In general, the treatment order for greatest horticultural value for all the parameters examined was: MLE + SA + GA > MLE + GA or SA individually > MLE alone > water-only control. Compared to other treatments, corms soaked in MLE + SA + GA had the earliest sprout time (3.7 days earlier than control), shortest production time (11 days earlier than control), tallest plant (159.5 cm), greatest number of leaves per plant (8.85 leaves), greatest maximum leaf area (66 cm^2^), highest SPAD reading (112) and photosynthetic activity (6.7 mmol m^−2^ s^−1^), longest spike length (91 cm), greatest number of florets per spike (20 florets), longest vase life (14.8 days), greatest N (1.53%), P (0.28%), and K (0.64%) concentrations, and largest corm diameter (4.68 cm) and mass (22.25 g). The highest total protein and proline concentrations were observed with the combined application of MLE + GA + SA. Our findings suggested that MLE either alone or in combination with other plant growth regulators not only increased the yield and quality of cut spikes, but also prolonged the vase life of cut gladiolus.

## 1. Introduction

Gladiolus (Iridaceae) is a monocotyledonous, herbaceous, perennial geophyte and cormous ornamental. Gladiolus is commercially planted by corms to produce cut flowers, and potted and garden ornamental plants. Cut gladiolus flowers highly rank in global floral markets with consistent demand throughout the year due to their unique ornamental value [1]. Thus, the demand for cut gladiolus continues to increase not only for local use, but also for export purposes in some countries [1,2]. However, maintaining cut flower quality is an important challenge for growers globally [3]. Successful cut flower production relies on several important factors, including a minimum production cost, and a short and predictable production time [4], as well as an extended post-harvest longevity [1].

Excessive use of synthetic fertilizers increase production costs, as well as increase potential environmental damage through nutrient leaching [5,6]. Therefore, there is increasing interest in finding sustainable solutions not only to minimize production costs and improving pre-harvest quality, but also for extending the post-harvest longevity of the bloom. In view of these challenges, the use of plant-derived biostimulants in horticultural crops has increased due to the economic, environmental, and biological advantages [7,8]. Among biostimulants, natural bioactive substances such as moringa (*Moringa oleifera*) leaf extract (MLE) have been shown to improve the pre- and post-harvest quality of horticultural crops [1,2,8]. The foliar application of MLE at various development stages of gladiolus not only improved the flowering quality, but also enhanced the post-harvest quality and longevity [1]. The advantageous effects of MLE are associated with the presence of a natural cytokinin, zeatin, vitamins, and minerals, which, when applied to plants, enhanced endogenous plant hormone biosynthesis, and ultimately enhanced growth and development [8].

The appropriate use of plant growth regulators (PGRs) in floriculture for lowering agronomic inputs is also a promising strategy. Salicylic acid (SA), a phenolic PGR, has been widely studied for its role in regulating plant growth and development, as well as the maintenance of postharvest longevity of cut flowers [9,10]. It is an endogenous regulator involved in plant defence activation that induces flowering [11]. Gibberellic acid (GA) is another well-known and important PGR that has been widely used in floriculture for promoting growth, flowering, quality, and post-harvest longevity [12,13,14].

In the present study, we evaluated the potential influence of MLE with or without SA and/or GA on the growth, physiology, flowering, and post-harvest longevity of gladiolus. The hypothesis was that soaking corms as a priming treatment with MLE with or without added SA and/or GA could enhance the growth, flowering quality, and post-harvest longevity of this important cut flower species.

## 2. Results

### 2.1. Days to 50% Sprouting

In gladiolus, the time to reach 50% corm germination significantly decreased (*p* < 0.0001) in response to all corm-soaking treatments compared to the water control (Table 1). The MLE treatment shortened the time to reach 50% germination by 25% (6.8 days) compared to the control (9.1 days). The addition of SA or GA with MLE did not shorten this time further over the treatment with MLE alone (6.5–6.8 days). However, the addition of both SA and GA to MLE significantly shortened the time to 50% germination by nearly another 1.5 days to 60% of the time needed for corms in the control treatment (Table 1).

### 2.2. Days to Harvest

There was a significant (*p* < 0.05) difference among corm soaking treatments regarding production time (days to harvest). Among the treatments, corms soaked in MLE + SA + GA had the shortest production time (71 days) relative to control (82.8 days) (Table 1).

### 2.3. Growth Parameters

Vegetative traits, i.e., plant height, number of leaves per plant, and leaf area were significantly (*p* < 0.0001) higher in treated plants than in control plants (Table 1). Corms soaked in MLE + SA + GA induced plant growth and development. This treatment showed the longest floral stem (159.5 cm), the highest number of leaves (8.9), and the greatest leaf area (65.9 cm^2^).

### 2.4. Leaf Mineral Composition

The effect of the treatments on macronutrient uptake was measured (Table 2). Corm soaking treatments (*p* < 0.0001) increased leaf concentrations of N, P and K in gladiolus. The MLE + SA + GA treatment had highest N (1.53%), P (0.28%), and K (0.64%) compared to all other treatments and the control (Table 2).

### 2.5. Physiological and Biochemical Parameters

Physiological parameters such as leaf greenness (SPAD) and photosynthetic rate showed significant difference among treatments (Table 3). Leaf chlorophyll non-destructively determined using SPAD and values ranged from 84.4 to 112.1. Control plants had the lowest value and MLE + SA + GA treatment had the highest value. The MLE-treated plants had an average SPAD value of 107.5. The addition of SA and GA had a synergistically positive effect. The positive effect was mainly induced by SA. The photosynthetic rate increased with the combination of treatments. As observed for leaf chlorophyll, the lowest value was found in the control plants (4.7 mmol m^−2^ s^−1^) and the highest values in the combined MLE + SA + GA treatment (6.69 mmol m^−2^ s^−1^) (Table 3).

No effect was observed in the protein concentrations of corm-soaked plants compared to the controls (Table 2). Soaking corm in MLE alone or in combination with SA or GA caused a slight increase in the proline concentration, while the greatest increase in proline concentration (27.04 µg g^−1^ FW) was observed in MLE + SA + GA in comparison to the control (22.70) (Table 2).

### 2.6. Corms Yield

A significant increase (*p* < 0.0001) in corm diameter and biomass was observed in response to all corm soaking treatments compared to the control (Table 3). As a general trend, the maximum increase in these parameters was observed in treatment MLE + SA + GA.

### 2.7. Ornamental Parameters

Corm soaking treatments had significant positive effects on flower quality parameters. Spike length, florets per spike, flower quality, and vase life were higher in the treatments compared to the control. Treatment MLE + SA + GA showed the highest spike length (91.08 cm), the highest number of florets per spike (20 florets), and the longest vase life (14.82 days) (Table 4). However, corm soaking treatments did not affect stem thickness, which ranged from 9.7 to 10 mm (Table 4).

## 3. Discussion

Gladiolus is an important floriculture crop widely grown as cut flowers or landscaping plants, because of its beautiful spikes and long period of flowering [1]. The high economic costs, and environmental and health-related issues of using agrochemicals are a serious challenge to growers [1]. Natural biostimulants can be innovative tools for improving the yield and quality of ornamental plants. They can be cheaper, safer, and more environmentally friendly than common agrochemicals. There is great interest in replacing synthetic agrochemicals with biostimulants [8]. Results clearly revealed that the application of MLE along with SA or GA can be used as a potential plant growth regulator to enhance the growth, physiology, and vase life of gladiolus, making this approach an economically viable option to replace synthetic fertilizers. The time to 50% corm sprouting and days to harvest were also shortened in response to all corm soaking treatments, but the most effective treatment was the combination of MLE with SA and GA.

Positive effects of GA have been observed in other bulb plants. In particular GA_3_ treatments were applied to nine tulip cultivars to break bulb dormancy. GA_3_ application increased sprouting, growth of floral stalks, and flowering in all cultivars studied [15]. Pre-planting application of GA_3_ to tulip bulbs resulted in earlier sprouting, higher sprouting percentage, higher photosynthetic rates and improved plant growth. The most efficient concentrations were 100 or 150 mg L^−1^ [16].

Faster corm sprouting and shorter times to harvest are important parameters for bulbous crops and have many advantages for growers, such as earlier and quicker returns on investment and minimizing the impact of unfavorable conditions that typically occur in late season. Uniform corm sprouting minimizes the labor and corm price expenses for replanting. Our results on these parameters agree with the findings of other researchers, e.g., Ahmad et al. [17] on freesia cut flowers and Nau [18] on calla lily. These results could be due to the presence of endogenous plant cytokinins (dihydrozeatin, isopentyladenine, and zeatin) in MLE [19,20]. Zeatin is a naturally and abundant endogenous cytokinin in plants that is vital for important biological processes, including cell division, cell elongation, anti-aging potential, growth enhancement, and protection [21,22]. In gladiolus, soaking treatment with GA showed a positive effect on breaking corm dormancy, promoting growth, and flowering [23]. Furthermore, MLE is an enriched source of micronutrients and various other growth promoting substances that enhancing plant growth [8].

Corm soaking or priming with plant growth regulators is a common practice in ornamental cut flower production. However, combined application of biostimulants with plant growth regulators has not been tested and their effect may vary with cultivars, crops, and dose. Our results showed an increase of plant development in response to all corm soaking treatments, particularly in MLE + SA + GA. The growth-promoting impact of MLE in combination with GA and SA alone and in combination may be due to the positive effect of these substances on cell division, enhancing cell and stem elongation, leaf expansion, plant growth, flowering, and senescence [24,25]. Bulb soaking in GA increased germination percentage, plant height, leaves per plant, and leaf area of tulip (*Tulipa gesneriana*) [16]. Soaking freesia corms in MLE improved corm budding percentage, growth, and ornamental traits as compared to the untreated corms [17]. With regard to GA, Bergman et al. [4] also reported a positive influence of GA application on the growth and ornamental traits of poinsettia ‘Renaissance Red’. Plant physiological traits are important indicators of improved plant function. SA is an important signal molecule that can activate different defense mechanism in plants [26]. The stress signal induced in the corm during priming could activate tolerance to biotic and abiotic stresses that helped the plants during the first stage of development.

In our study, corm soaking treatments increased the SPAD value and photosynthetic activity of gladiolus during cultivation. The increase in leaf area and chlorophyll concentration can explain the higher photosynthetic rate and better plants performance. At the biochemical level, these increases likely mean higher sugar production and biomass accumulation with a positive effect on growth and flower development. An increase in chlorophyll concentration in biostimulant-treated plants is a common physiological marker for the activation of primary metabolism [27]. A potential reason for an increase chlorophyll and photosynthetic activity may be the presence of substances promoting chlorophyll biosynthesis [28] in moringa leaves, including carotenoids and minerals [29,30]. GA is also an important plant hormone involved in the chlorophyll biosynthesis and stem elongation [31]. Increased chlorophyll concentration following MLE application correlates with a delay in leaf senescence due to the presence of ascorbate, zeatin, and potassium in MLE [30]. An increase in mineral concentration and plant physiological traits may result in improved ornamental quality and vase life. In a similar study, soaking gladiolus corms in 5% MLE resulted in improved ornamental quality and vase life [17]. The increase in leaf proline concentration in the combined treatment suggested that this treatment could act in the reduction of the stress condition of the plant and increase the crop performance.

## 4. Materials and Methods 

### 4.1. Solutions 

Fresh mature moringa leaves (100 g) were harvested from a mature plant grown at the experimental area of the Department of Forestry, Range and Wildlife Management at the University of Agriculture, Faisalabad, Pakistan (31°25′30′′ N 73°04′15′′ E). After harvesting, the leaves were washed with tap water to remove dust and debris. After drying in the shade for 1 h, leaves were stored at −5 °C overnight. Stored leaves were pressed and filtered twice using cheese cloth and Whatman filter paper no 1 [32,33]. The filtered extract was centrifuged at 8000× *g* for 15 min to remove fine debris. The supernatant was diluted with 20 volumes of distilled water to make a 5% (*v/v*) MLE working solution. MLE had the following mineral composition on a dry weight basis: 6.70 mg g^−1^ Mg^2+^, 28.0 mg g^−1^ Ca^2+^, 0.75 mg g^−1^ Na^+^, 25.1 mg g^−1^ K^+^, 8.10 mg g^−1^ P, 0.84 mg g^−1^ Mn, 1.60 mg g^−1^ Fe^2+^, 0.27 mg g^−1^ Zn+, and 0.14 mg g^−1^ Cu^2+^ [30]. Furthermore, the extract also contained soluble phenolics (6.20 mg g−1), proline (21.00 mg g−1 DW), total carotenoids (3.10 mg g^−1^), ascorbic acid (242.40 mg 100 g^−1^ FW), amino acids (106.20 mg g^−1^), and soluble sugars (248.7 mg g^−1^). The plant hormones present in MLE were indole-3 acetic acid (0.83), GA (0.74), zeatin (0.96), a cytokinin and abscisic acid (0.29) [30].

GA (50 mg L^−1^; Sigma Aldrich Corp., St. Louis, MO, USA) and SA (50 mg L^−1^; Sigma Aldrich Corp., St. Louis, MO, USA) were dissolved in 5% (*v/v*) MLE either alone [(MLE 5%), (MLE 5% + GA 50 mg L^−1^) or (MLE 5% + SA 50 mg L^−1^)] or in combination (MLE 5% + GA 50 mg L^−1^ + SA 50 mg L^−1^). Corms were soaked in these solutions or distilled water as a negative control for 24 h at 20 °C in darkness.

### 4.2. Experimental Setup

A pot experiment was conducted at the Lalazar Nursery Area, University of Agriculture, Faisalabad (31°25′36′′ N 73°04′15′′ E). Healthy and equal-sized corms (mean weight 4.5 g; mean diameter 10 cm) of gladiolus (*Gladiolus grandiflorus*) ‘Nova’ were surface sterilized with 5% (*v/v*) NaOCl for 6 min, washed with double-distilled water and left to dry in shade. Corm scales were removed and soaked for 24 h in freshly prepared solutions of 5% MLE, 5% MLE + SA, 5% MLE + GA and 5% MLE + SA + GA. After soaking, corms were dried in the open air in shade at ambient temperature and planted in soil-filled plastic pots. The bulbs were planted on 20 Sep, 2019. The characteristics of the soil were: 0.655% organic matter, pH 7.2; EC (dSm^−1^) 0.92; 0. 028 % N; 9.8 mg kg^-1^ available P and 203 mg kg^−1^ exchangeable K.

### 4.3. Plant Vegetative and Reproductive Growth

The time to 50% corm sprouting (sprouted = the young buds reach one centimeter in length) and days to production harvest (from planting to complete flowering) were counted. Plant height, total number of leaves per plant, and leaf area (LICOR-3000C Portable Area Meter; LI-COR, Lincoln, NE, USA) were measured. Inflorescence/spike length, number of florets per spike, and stem thickness were measured. At the time of spike harvest, two lower leaves were left intact to support corm development [1]. On maturity, corms were uprooted and washed with tap water. Corm mass and diameter were measured.

### 4.4. Vase Life Determination 

Flower spikes free from visual defects were harvested with a sharp steel knife during the morning hours at the commercial maturity stage, i.e., uniformity in size and quality, 2 to 3 florets per spike in color break stage. After harvest, the cut spikes were placing upright in a bucket partially filled with water and moved to the laboratory within 15 min. Moisture loss was minimized by shrouding the cut flowers with soft plastic film during the move. In the laboratory, spikes were re-cut at approximately 90 cm length under water to minimize air emboli and vascular blockage. One spike per vase were placed in 200 mL vases filled with deionized distilled water and covered with aluminum foil to prevent evaporation. Vases were kept at 25 ± 2 °C, relative humidity 60 ± 5%, under white fluorescent lamps with a 14 h light 10 h dark photoperiod. A total of 20 cut flower spikes were used per treatment to assess vase life. The end of vase life was defined as the number of days from harvest until 60% of the flower petals had wilted or had no aesthetic value due to loss of turgidity or color changes. Data were recorded daily up to 15 days.

### 4.5. Leaf Physiological Parameters and Mineral Concentrations

Leaf greenness index measurements (SPAD 502, Konica Minolta, Japan) were taken from base, middle and tip of fully expanded leaves at the phenological stage of full flowering of plants. Net photosynthetic carbon assimilation rate (µmol m^−2^ s^−1^) was measured at the phenological stage of full flowering of plants on three mature, healthy, and fully expanded sunlit leaf blades from six plants per treatment between 9.00 am and 11.00 am using an infrared gas analyzer (LI-COR 6400, LI-COR, Lincoln, NE, USA) set at 400 μmol m^−2^ s^−1^ CO_2_ and a flow rate of 300 μmol m^−2^ s^−1^. Leaf nitrogen (N) concentration was determined by the Kjeldhal method according to Jackson [34]. Leaf phosphorus (P) and potassium (K) concentrations were determined according to Chapman and Pratt [35].

### 4.6. Total Soluble Proteins and Leaf Free-Proline Contents

Total soluble protein was determined at 595 nm using bovine serum albumin as a standard [36]. A ninhydrin-based method was used to measure leaf free-proline concentration [37]. Briefly, 0.5 g fresh leaf material was extracted in 10 mL of 3% (*w/v*) sulfo-salicylic acid. Afterwards, 2.0 mL of the filtered solution was added to 2.0 mL of acid ninhydrin (1.26 g ninhydrin + 20 mL 6 M ortho-phosphoric acid + 30 mL glacial acetic acid) and 2.0 mL of glacial acetic acid. Samples were incubated at 80 °C for 60 min and immediately moved to an ice bath to terminate the reaction. Toluene (4.0 mL) was added to the solution and vigorously mixed by vortexing for 30 s. The chromophore-containing toluene was separated from the aqueous phase and its absorbance read at 520 nm.

### 4.7. Experimental Design and Statistical Analysis

The experiment was performed in a completely randomized fashion with twenty plants per treatment and a total 5 treatments (MLE, MLE + GA, MLE + SA, MLE + SA + GA, and control) for a total of 100 plants. Data were analyzed via ANOVA. Means were separated using all comparisons through Tuckey’s HSD test (*p* ≤ 0.05) (statistix 8.1 Analytical Software, Tallahassee, FL, USA).

## 5. Conclusions

Agricultural systems are evolving towards more economic and environmentally friendly cultivation strategies. Biostimulants are greatly enhancing crop performance, thereby reducing agrochemical inputs, without compromising the quality and yield of crops. In the present work, a pre-planting treatment of soaking corms in combinations MLE and plant growth regulators (GA and SA) has been demonstrated to be an inexpensive and efficient system for enhancing plant performance. The results indicated that the addition of bioactive molecules in combination with plant growth regulators has a synergistic effect on cut gladiolus growth and development. At a practical level, these results allow for the modulation of the crop growth cycle such that the combined MLE treatment can also be exploited in the programming of flowering, which is extremely important to the ornamental sector. These encouraging results open the possibility for further studies focused on optimizing the concentration, timing, and mode of application of MLE alone or in combination with various plant growth regulators.

## Figures and Tables

**Table 1:** Effect of 24 h corm soaking on time to 50% germination, production time, and vegetative traits of gladiolus (*Gladiolus grandiflorus* cv. “Nova”).

* Treatments	50% Germination (Days)	Production Time (Days to Harvest)	Plant Height (cm)	Leaves per Plant (Number)	Leaf Area (cm^2^)
Control	9.1a ^1^	82.8a	134.4c	7.4c	59.5c
MLE	6.8b	75.0b	148.8b	8.5b	62.0bc
MLE + SA	6.6b	73.0c	151.9b	8.6ab	63.6ab
MLE + GA	6.5b	72.0d	151.5b	8.6ab	62.2bc
MLE+SA + GA	5.4c	71.0e	159.5a	8.8a	65.8a
^**^ ANOVA *p* value	<0.0001	0.0000	<0.0001	<0.0001	<0.0001

^1^ Values are means (*n* = 20). Different letters indicate significant differences. Differences between means were compared by Tukey’s HSD post-hoc test using all pairwise comparisons (*p* < 0.05). * MLE (5% Moringa leaf extract), MLE + SA (5% Moringa leaf extract + salicylic acid), MLE + GA (5% Moringa leaf extract + gibberellic acid), MLE + SA + GA (5% Moringa leaf extract + salicylic acid + gibberellic acid). ^**^ ANOVA: Analysis of variance (One way).

**Table 2:** Effect of soaking corms (24 h) in different treatment solutions on leaf concentrations of macro nutrients (N, P and K%) and leaf biochemical parameters (total soluble protein and proline) in gladiolus (*Gladiolus grandiflorus* cv. “Nova”).

* Treatments	Leaf N (%)	Leaf P (%)	Leaf K (%)	Total Soluble Protein (mg g ^−1^ FW)	Proline (µg g ^−1^ FW)
Control	0.94e ^1^	0.15d	0.42e	16.40ab	22.70c
MLE	1.37d	0.24c	0.51d	14.84b	23.96b
MLE + SA	1.42c	0.26b	0.59b	15.78ab	23.06c
MLE + GA	1.45b	0.25c	0.56c	16.76a	23.94b
MLE + SA + GA	1.53a	0.28a	0.64a	17.18a	27.04a
^**^ ANOVA Significance	<0.0001	<0.0001	<0.0001	0.0041	<0.0001

^1^ Values are means (*n* = 20) and differences between means were compared by the post-hoc tukey test using all pairwise comparison at *p* = 0.05. Mean pairs followed by different letters are significantly different. * MLE (Moringa leaf extract 5%), MLE + SA (Moringa leaf extract 5% + Salicylic acid), MLE + GA (Moringa leaf extract 5% + gibberellic acid), MLE + SA + GA (Moringa leaf extract 5% +Salicylic acid+ gibberellic acid). ** ANOVA: Analysis of variance (One way).

**Table 3:** Effect of soaking corms (24 h) in different treatment solutions on corm size and plant physiological traits (SPAD, Photosynthesis rate) in gladiolus (*Gladiolus grandiflorus* cv. “Nova”).

^*^ Treatments	Corm Diameter (cm)	Corm Mass (g)	Leaf Greenness (SPAD)	Photosynthesis Rate (mmol m^−2^ s^−1^)
Control	3.2e ^1^	13.3d	84.4c	4.6d
MLE	4.3d	16.6b	107.5b	5.6c
MLE + SA	4.5b	16.7b	110.0ab	6.0bc
MLE + GA	4.4c	15.6c	108.1ab	6.2ab
MLE + SA + GA	4.6a	22.2a	112.1a	6.6a
** ANOVA Significance	<0.0001	<0.0001	< 0.0001	<0.0001

^1^ Values are means (*n* = 20) and differences between means were compared by the post-hoc tukey test using all pairwise comparison at *p* = 0.05. Mean pairs followed by different letters are significantly different. * MLE (Moringa leaf extract 5%), MLE + SA (Moringa leaf extract 5% + Salicylic acid), MLE + GA (Moringa leaf extract 5% + gibberellic acid), MLE + SA + GA (Moringa leaf extract 5% +Salicylic acid + gibberellic acid). ** ANOVA: Analysis of variance (One way).

**Table 4:** Effect of corm soaking (24 h) in different treatment solutions on the ornamental traits (spike length, florets per spike and vase life) of gladiolus (*Gladiolus grandiflorus* cv. “Nova”).

^*^ Treatments	Spike Length (cm)	Florets per Spike (No)	Vase Life (Days)	Stem Thickness (mm)
Control	86.2b^1^	15.2b	11.3c	9.7a
MLE	92.3a	18.7a	13.0b	9.9a
MLE + SA	93.4a	19.4a	12.4bc	10.0a
MLE + GA	89.2ab	18.6a	13.7ab	9.8a
MLE + SA + GA	91.0a	20.0a	14.8a	9.8a
** ANOVA Significance	0.0002	<0.0001	<0.0001	0.2567

^1^ Values are means (*n* = 20) and differences between means were compared by the post-hoc tukey test using all pairwise comparison at *p* = 0.05. Mean pairs followed by different letters are significantly different. * MLE (Moringa leaf extract 5%), MLE + SA (Moringa leaf extract 5% + Salicylic acid), MLE + GA (Moringa leaf extract 5% + gibberellic acid), MLE + SA + GA (Moringa leaf extract 5% + Salicylic acid + gibberellic acid). ** ANOVA: Analysis of variance (One way).
